# Dr. Abolghasem Bahrami (1894–1950): Physician, Pasteurian, and a Pioneer of Microbiology and Public Health Planning in Iran

**DOI:** 10.52547/ibj.26.2.91

**Published:** 2021-12-06

**Authors:** Fariborz Bahrami, Ehsan Mostafavi

**Affiliations:** 1 Department of Immunology, Pasteur Institute of Iran, Tehran, Iran;; 2 Department of Epidemiology and Biostatistics, Research Centre for Emerging and Reemerging Infectious Diseases, Pasteur Institute of Iran, Tehran, Iran

**Keywords:** History of medicine, Iran, Microbiology, Pasteur Institute of Iran, Public health

## Abstract

Dr. Abolghasem Bahrami was among the generation of Iranian scientists in the early twentieth century who gained most of their knowledge through resources available inside the country. Educated at Dar-ul-Funun Medical School, he was a physician with a great talent in learning, especially self-teaching natural sciences and European languages. He joined the Pasteur Institute of Iran (IPI) at the early days of its foundation and became an integral contributor to this institution during the first twenty-five years of its mission. One of his first assignments at IPI was to help initiating an anti-rabies department by bringing back the rabies vaccine and its manufacturing equipment from Institut Pasteur of Paris. During his IPI years, aside from managerial tasks, he actively participated in upgrading the medical treatments and protocols used for controlling many infectious diseases. He functioned twice as the provisional director of IPI (1925-1926 and 1937-1946) and is considered as the first Iranian director of the Institute. Meanwhile, Dr. Bahrami was a significant contributor to the public health system and assumed several responsibilities such as Chief Quarantine Medical Officer, Chief of Public Health, and the Head of Public Health Administration, in order to improve public health planning throughout the country.

## Introduction

**Early life and education**

Based on resources such as Bahrami family genealogy and facts remained from documented lectures of Iranian medical historian, Professor Mahmoud Najmabadi (1903-2000), Abolghasem Bahrami was born in Tehran in 1894 into a distinguished family of mostly educated high-ranking civil servants, military officers, technocrats, and physicians. Members of the Bahrami family were originally from the city of Tafresh who had migrated to the new capital of the Qajar dynasty, over a span of a hundred years, due to their involvements in the country’s bureaucratic hierarchy^[^^1^^]^. Abolghasem’s father, Dr. Mirza Abolhassan Khan, was the personal physician of Ala-al-Dowleh, the Governor of Tehran and the Minister of the Royal Court during Nasser al-Din Shah’s reign. Since early childhood, Abolghasem followed the footsteps of his father and elder brother, Dr. Alireza Bahrami, in pursuing an education in science and medicine. After his elementary studies, he first attended Tehran’s German School and later continued his education at the renowned Dar-ul-Funun Medical School, one of the first institutions of higher education in the country. In addition to receiving medical training by his father and attending courses in medicine and anatomy, he studiously pursued his studies in Persian literature, German and French languages, geography, mathematics, and experimental sciences at Dar-ul-Funun. In 1919, at the age of 25, he became an instructor in a college, called Dar-ul-Moalemin Markazi, for training teachers^[^^2^^]^.

**Years at the Pasteur Institute of Iran (IPI)**

The long-neglected Iranian medical capabilities and infrastructure were at best chaotic in the wake of the early-twentieth century upheavals caused by the First World War and, most notably, the 1918–1919 influenza pandemic and its massive casualties. Moreover, the outbreaks of infectious diseases such as plague, cholera, and malaria across the country had placed the population in a dire situation. Consequently, the Iranian government was forced to recognize an urgent need for an organization to combat infectious diseases and to repair its outdated public health system. Due to the long history of interactions between Iranian elites and French physicians, such as the famous medical doctor of the royal court, Dr. Joseph Désiré Tholozan (1820–1897), and the influence of French academia in shaping modern medicine in Iran, France and its well-known biomedical research organization, Institut Pasteur of Paris, seemed to be an obvious choice in the quest for improving the public health system. The initial contacts were made by an Iranian medical delegation under the direction of Mohammad Ali Foroughi (Zoka-al-Molk; 1875–1942) with Institut Pasteur in Paris in October 1919. Three months later, Prince Firouz Mirza Nosrat-ed-Dowleh III (1889–1937), the Minister of Foreign Affairs and the head of the Persian delegation to the Paris Peace Conference reached an agreement with Institut Pasteur, which laid the groundwork for the establishment in Iran of the tenth worldwide branch of this renowned institution^[^^3^^,^^4^^]^. Pierre Paul Émile Roux (1853–1933), the Institute’s Director at that time, dispatched a bacteriologist, Dr. Joseph Mésnard (1886–1950), to Tehran in 1920 to oversee the establishment process^[^^4^^]^. 

There are two published memoires of Dr. Abolghasem Bahrami’s later junior colleague and friend, Dr. Mahdi Ghodssi (1900-1991), in Persian, namely “*The History of the fifty years of the services of the Pasteur Institute of Iran” *(1971)^[^^5^^]^ and “*The memories of school days and years of service at the Pasteur Institute of Iran” *(2016)^[^^6^^]^, which have shed much light on this period of Abolghasem Bahrami’s life. Upon Dr. Mésnard’s arrival in Tehran, Abolghasem Bahrami was hired to assist him due to his strong scientific background and advanced knowledge of the French language^[^^5^^]^. Before this appointment, he was a teacher of Natural Sciences at his alma mater (Dar-ul-Funun) and also a lecturer of Zoology and Comparative Anatomy at the Higher College of Medicine in Tehran. After being hired at IPI, Abolghasem Bahrami obtained his medical degree, coinciding with the coup d'état of 1921 in Tehran, which led to the eventual downfall of the Qajar dynasty. Dr. Ghodssi has emphasized that Abolghasem Bahrami was gifted in learning, especially learning European languages, which enabled him to self-teach using French, English, German, and Italian resources. Thanks to this advantage, he completed the medical school curriculum and achieved his degree in medicine by passing the final examinations without actually attending all the requisite courses. Moreover, being brought up and trained in a family of physicians, he was able to successfully practice medicine during his professional life without any internship or externship trainings in hospitals^[^^6^^]^.

The initial headquarters of IPI, designated by Dr. Mésnard in 1921, was later moved to a permanent campus on a 10,000 square meter prime lot in central Tehran. The land was generously offered with a substantial cash payment by Prince Abdol-Hossein Farman Farma (1852–1939), the father of Prince Firouz Mirza, as part of a religious endowment. This gracious donation and additional state contributions allowed for the first buildings of IPI to be constructed at a later date^[^^7^^]^. One of the early assignments of Dr. Bahrami at IPI was focused on rabies. Due to the lack of anti-rabies treatments in Iran at that time, he was dispatched for a few months to Paris in 1922 to bring back the rabies vaccine, which had been developed by Institut Pasteur of Paris, along with its manufacturing equipment via a long land journey. In Paris, Dr. Bahrami received training as a Fellow at the laboratory of Professor René Legroux (1877–1951), and on his return, he and Dr. Mésnard together established an anti-rabies department at IPI^[^^8^^]^. Moreover, in 1925, he was sent to Italy along with two other physicians by the government to study malariology^[^^9^^]^. 

**Fig. 1:**
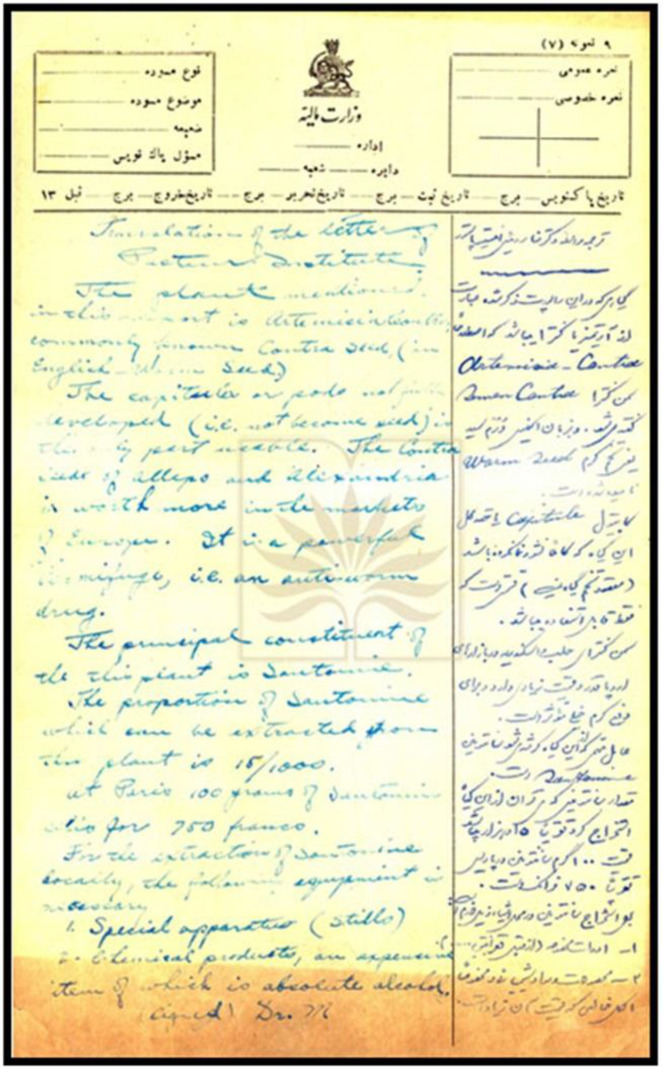
A sample of Mésnard’s handwriting in 1923, describing a pharmaceutical plant with its translation in Persian (National Library and Archives of Islamic Republic of Iran public records)

Until Dr. Mésnard’s departure to France in 1925, Dr. Bahrami functioned as his deputy and translator (Fig. 1). He extended these duties to Dr. Mésnard’s successor, Dr. Jean-François Kérandel (1873–1934). The relatively long apprenticeship of Dr. Bahrami with Dr. Mésnard helped him to acquire a solid background in microbiology, which is reflected in his later publications and activities. During the one-year gap between Dr. Mésnard’s departure and the arrival of Dr. Kérandel in September 1926, the provisional directorship of the Institute was assigned to 

**Fig. 2:**
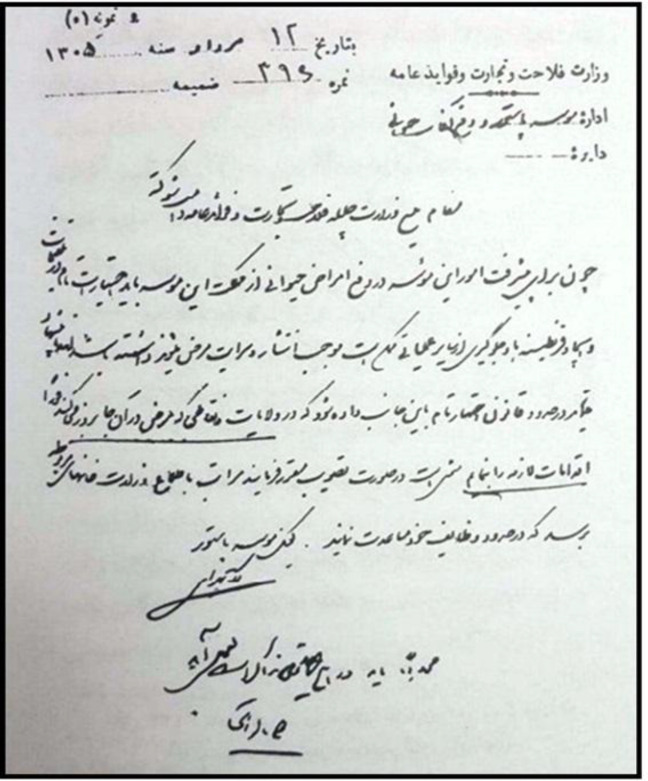
A handwritten request signed by Dr. Bahrami  (in 1926), as the provisional director of IPI, to the Ministry of Agriculture, Commerce and Public Services for having full authorization to take action for stopping diseases throughout the country (National Library and Archives of Islamic Republic of Iran public records)

Abolghasem Bahrami; hence, he is considered the first Iranian director of IPI (Fig. 2). 

**Fig. 3:**
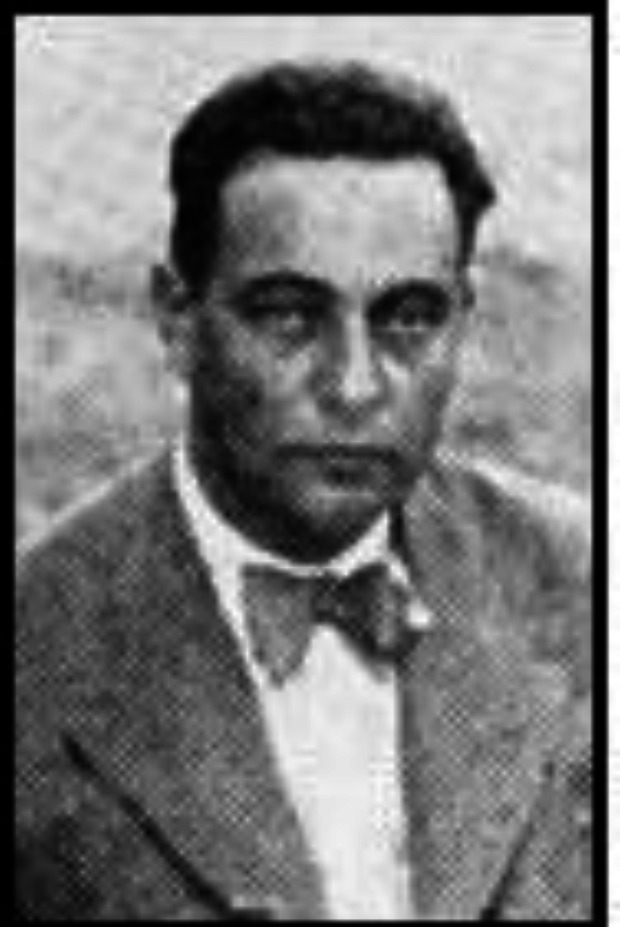
Portrait of Dr. Abolghasem Bahrami, circa 1935^[^^5^^]^

Following serving as the director of the Institute for eight years, Dr. Kérandel lost his life due to pneumonia in 1934, and Dr. Hossein Mashouf assumed the provisional directorship of the Institute. In September 1935, Professor Legroux, who had presided over the creation of IPI for the previous fifteen years, came to Iran and was elected as the Honorary Director of the Institute. As Dr. Ghodssi recounts in his memoir, during Professor Legroux’s periodic visit to Iran in 1937, Dr. Bahrami (Fig. 3) was appointed to the provisional directorship of the Institute for the second time. Due to the onset of the Second World War and occupation of France by Nazi Germany, which disrupted the IPI contacts with Institut Pasteur of Paris, his second provisional directorship lasted till 1946^[^^6^^]^. 

Although Iran was a neutral country during the Second World War, the ensued calamities such as famine, food shortages, infectious diseases, and foreign occupation did not spare the country. During the war time, Dr. Bahrami managed the Institute with the help of his junior assistants, namely Dr. Mahdi Ghodssi, Dr. Mahdi Zol-Riasatein, and Dr. Sadegh Moghadam. Educated female staff, including Ms. Ezzat Marashi, Ms. Aghdas Partovi, and Dr. Robabeh Kianoori, to name a few, had also joined the Institute by this time. 

**Fig. 4:**
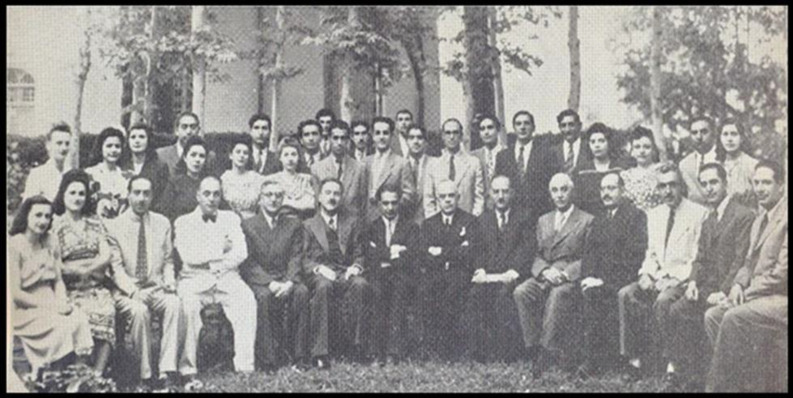
The high-ranking French Pasteurians and the personnel of IPI, celebrating the 25^th^ anniversary of its establishment; Tehran, August 1946. On one of his last days at IPI, Dr. Bahrami is the fourth seated person from the left on the first row. Dr. Baltazard is the third seated person from the right on the first row.^[^^5^^]^

After the end of the war and liberation of France, the contacts between IPI and Institut Pasteur of Paris were reestablished. In January 1946, Dr. Marcel Baltazard (1908–1971), a French physician and medical investigator who had previously conducted medical research at the Pasteur Institute of Morocco, arrived in Tehran on a temporary appointment. Dr. Baltazard was hosted and assisted by Dr. Bahrami on the IPI campus where he briefly studied the ongoing activities related to rabies and tetanus. Meanwhile, he prepared a new contract between the Iranian government and Institut Pasteur of Paris, which was eventually signed by the Iranian officials and invited high-ranking Pasteurians, including Professor Legroux^[^^6^^]^. According to a memoir of Dr. Ghodssi, who had close relationships with Professor Legroux, the initial intention of the professor was to retain the directorship of Dr. Bahrami with the new contract since he had high confidence in capabilities of Iranian scientists in general, and Dr. Bahrami who had managed IPI for the previous nine years, in particular. However, due to previous conflicts between Dr. Bahrami and an influential cabinet minister, or perhaps other considerations, the Pasteurian delegation was encouraged not to nominate Dr. Bahrami as the next director of the Institute^[6]^. In August 1946, after ratifying an updated charter of IPI, Dr. Baltazard was hence appointed as the next director of IPI and held this position for the next twelve years. The French delegation’s visit coincided with celebrations of the twenty-fifth anniversary of the Institute (Fig. 4). Following Dr. Baltazard’s appointment, Dr. Bahrami left IPI for good^[^^5^^]^.

**Publications**

**Fig. 5:**
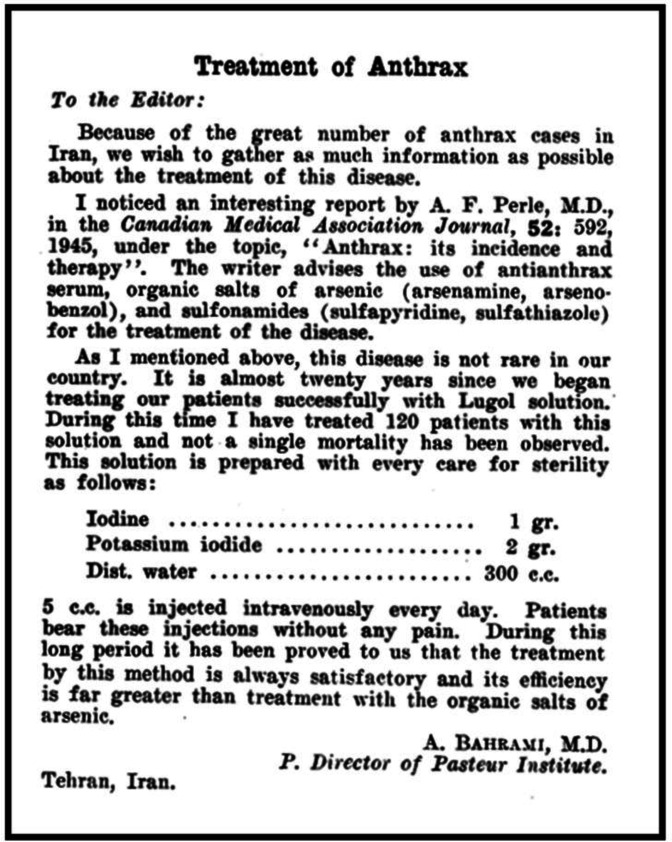
A letter to the editor published in Canadian Medical Association Journal in 1946^[^^10^^]^

**Fig. 6:**
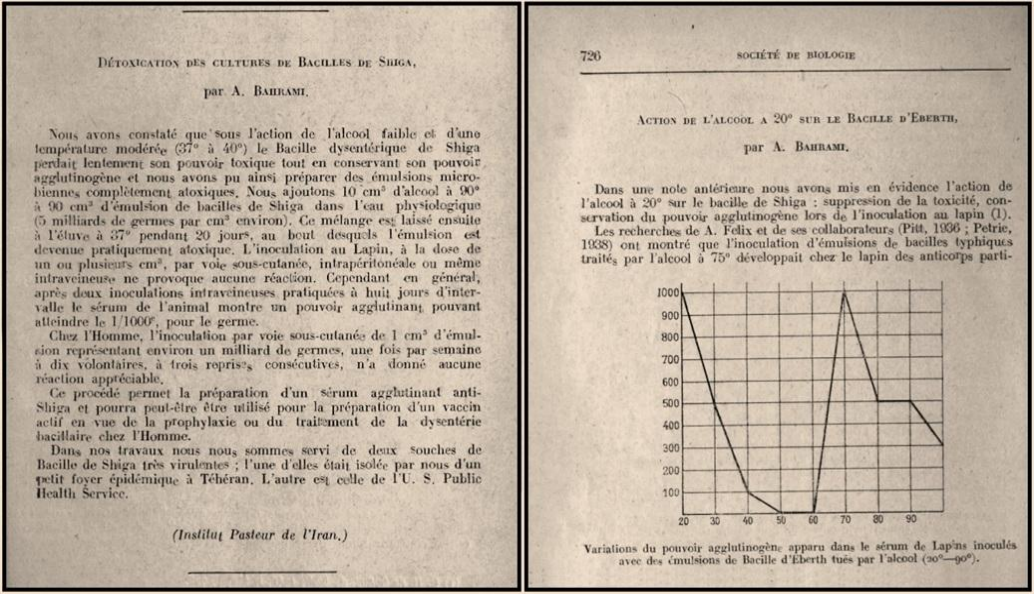
Two articles by Dr. Bahrami published in Comptes rendus des séances de la société de biologie et de ses filiales in October 1946 (BnF Gallica public records).

According to our current knowledge, four publications were penned by Dr. Abolghasem Bahrami in international journals during 1946–1947, his survival period. One of them is a letter to the editor in English, titled “*Treatment of Anthrax*”, published in the Canadian Medical Association Journal in June 1946 (Fig. 5). This document shows that he was acquainted with the medical journals of his time. In this letter, based on his experiences for the successful treatment of 120 anthrax patients, he offers a better protocol for the treatment of this disease compared to what was suggested by another physician in the same journal, a year earlier^[^^10^^]^. The other three documents are in French. Two were published back-to-back in a French journal, named Comptes rendus des séances de la société de biologie et de ses filiales, in 1946. The first is titled “*Détoxication des cultures de bacilles des Shiga*” [Detoxification of *Shiga *bacillus cultures], in which Dr. Bahrami describes his experiments in disinfecting the virulent *Shiga* bacillus with weak alcohol at two different temperatures. The other article is titled “*Action de l'alcool à 20*^o^* sur le bacille d'Eberth*” [Effects of 20^o^ alcohol on *Eberth *bacillus] and describes his experiments on nine different concentrations of alcohol on the causative bacterium of typhoid fever with a chart that depicts different agglutination powers of the pathogen with the tested alcohols of different strengths (Fig. 6). The fourth article, titled “*Un colorant du remplacement du Giemsa*” [A substitute for Giemsa stain]^[^^11^^]^, was published in Bulletin de la société de pathologie exotique in 1947. Once again, in this paper, he offers a better method, in this case for microbiological staining, than the established protocol, based on his own experiments. The importance of the above publications would be more evident when they are put in the context of socioeconomic and political conditions of the country during that era. In mid 1940s, Iran was still struggling against the miseries of the Second World War and the foreign occupation. Moreover, means of communications and transportation were disrupted across the globe, while the essentials for normal life were unsettled by many shortages. Under these conditions, one ought to appreciate the rare spirit of scientific perseverance of Dr. Bahrami for an Iranian scientist of that epoch, for trying to have access to the international scientific literature and to interact with his contemporary scientific community. Dr. Bahrami also authored textbooks in Persian. A document issued by the Ministry of Culture (The Bureau of Publications) 

**Fig. 7:**
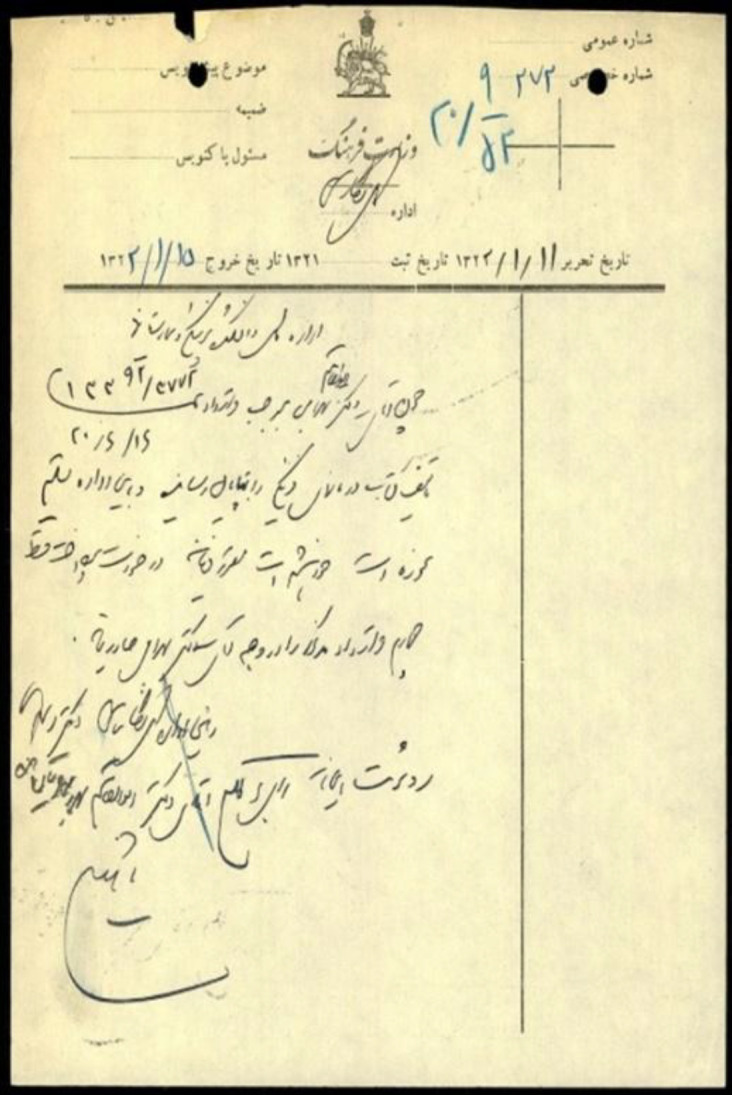
A payment order issued by the Ministry of Culture to the general directorate of the Medical School and the hospitals on 5 April 1943, for the fourth instalment of contract No. 13392-3772/23-Aug-1941, for authoring a book titled “*Darman-haye fiziki*” [Physical treatments] by Dr. Abolghasem Bahrami due to completion of the writing (National Library and Archives of Islamic Republic of Iran public records)

indicates that Dr. Abolghasem Bahrami completed and submitted a book titled “*Darman-haye fiziki*” [Physical treatments] in 1943 within two years after signing a contract with the Ministry (Fig. 7). Unfortunately, no copy of this book has so far been recovered by the authors of the present article. Furthermore, a textbook in Persian titled “*Tajzieh edraar*” [Urinalysis] of 199 pages containing diagrams, tables, and protocols based on Dr. Bahrami’s manuscript and notes was published posthumously by Tehran University Press in 1956^[^^12^^]^. 

**Fig. 8:**
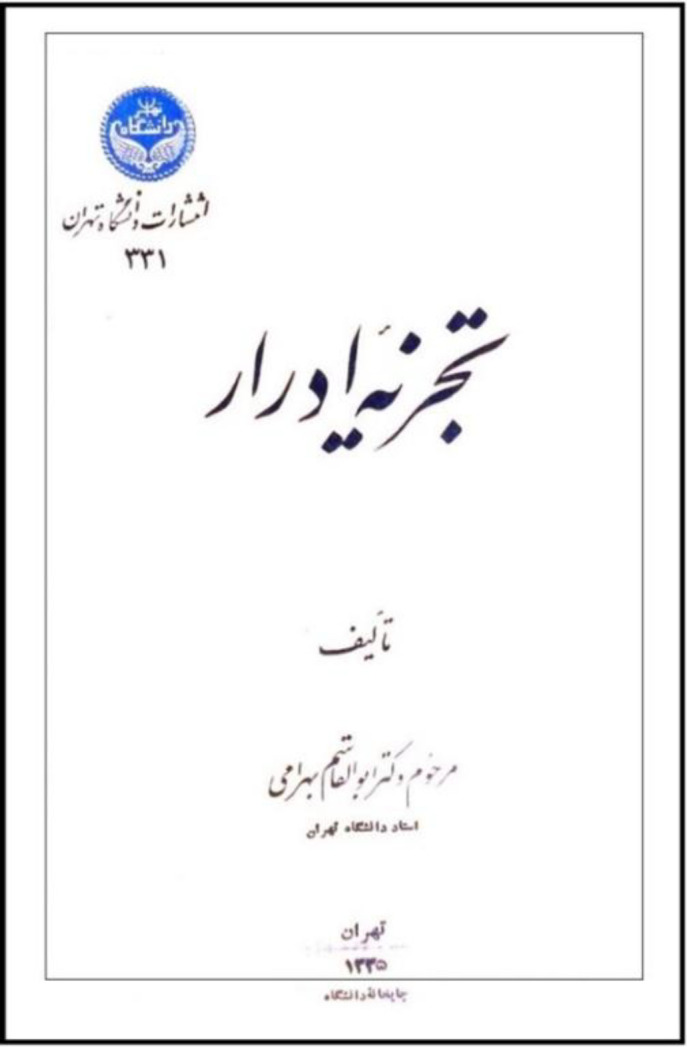
Front page of a textbook authored by Dr. Bahrami and published posthumously by Tehran University Press in 1956, titled “*Tajzieh edraar*” [Urinalysis].

The manuscript was originally inspired based on translations of French, English, German and Italian resources of the time on the subject matter to which Dr. Bahrami had added his own notes and interpretations. Six years after Dr. Bahrami’s death, the manuscript was prepared for publication by Dr. A. Attaei, Dr. M.A. Kazemi, and Dr. S.A. Alavi Naeini who were his colleagues at the Department of Veterinary Medicine of Tehran University. Aside from describing methods to be used for analyzing a patient’s urine for medical evaluation, the textbook meticulously elaborates physicochemical interactions that alter the constituents of urine based on the latest available scientific knowledge and methodology (Fig. 8). 

**Public health services**

**Fig. 9:**
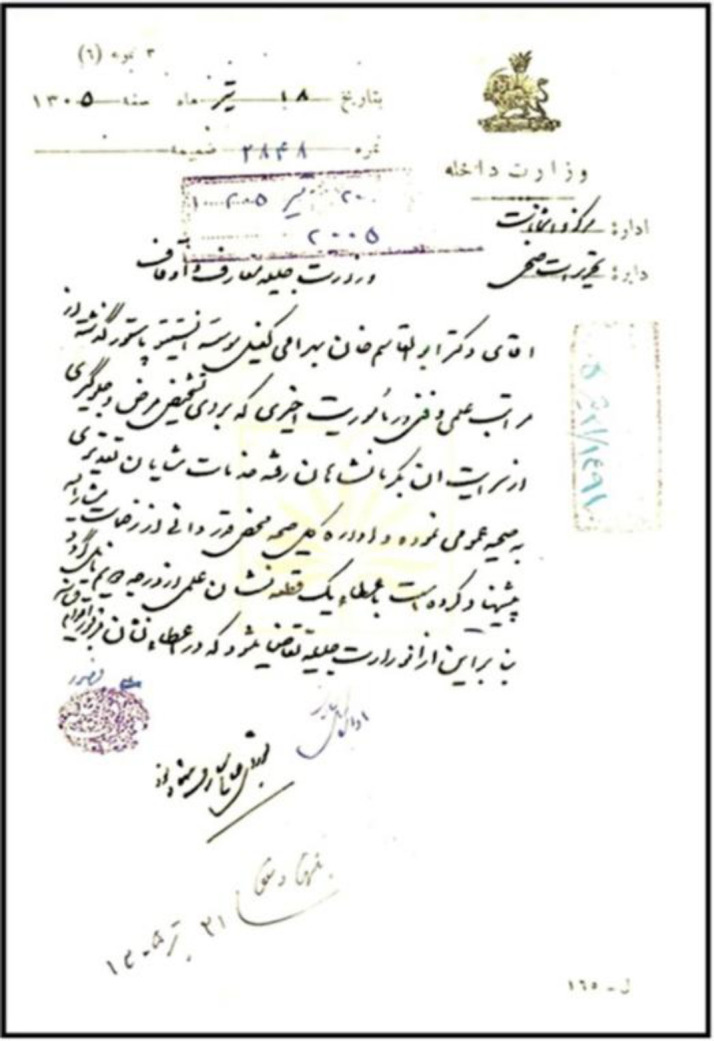
A letter from the Ministry of Interior Affairs, dated 10 July 1926, to the Ministry of Education and Endowments, requesting a second-degree Medal of Science to be bestowed to Dr. Abolghasem Bahrami, the provisional director of the Pasteur Institute of Iran, in appreciation for his outstanding public health services during his mission in Kermanshahan region (National Library and Archives of Islamic Republic of Iran public records)

Dr. Bahrami was assigned to public health missions in several provinces of the country such as Kermanshahan in the West (Fig. 9), Bushehr and Shiraz in the South, and Chalus, Noshahr, and Tonekabon in the North. In 1928, in an attempt to abolish capitulations to foreign powers, the government of Iran decided to take over the quarantine service in Persian Gulf ports from Britain and replaced the British medical staff in the southern port cities with the Iranian ones^[^^13^^]^. Dr. Bahrami was one of these physicians and was appointed as a Chief Quarantine Medical Officer in Bushehr for four years under the harsh living conditions of the port at that time. According to the British physician and historian, Dr. Cyril Elgood, in his renowned book, “*A Medical History of Persia*”, the young Dr. Bahrami was keen and energetic in carrying out his duties in Bushehr, spoke French well and had a good knowledge of bacteriology^[^^14^^]^. After Bushehr, he went to Shiraz and became the Chief of Public Health in Fars province^[^^15^^]^.

According to an article called, “*About* *Reconstruction of Noshahr*”, published in Khaterat-e-Vahid magazine in Persian (volume 10, issue 8, 1972, pp. 62-65), Dr. Bahrami, the deputy director of IPI, was missioned by the government to the Caspian Sea coasts in the North in 1933 to bring the outbreak of malaria under control in Noshahr and Chalus cities. Dr. Bahrami went to the region, accompanied by a well-equipped group and controlled malaria by source reduction. To eliminate malaria-carrying mosquitoes and their breeding sites, he distributed free insecticide and hand pump sprayers to the local villagers and removed the standing waters or covered them with petroleum in remote villages and jungles, during muleback ridings in which he personally participated. He had a great success in this mission within a short period of time and, in addition, managed to establish a 50-bed hospital in Chalus. Then by a government directive, he handed over a hospital built by a Dutch company for its employees in Noshahr to the national public health authorities. Before being summoned to Tehran to assume his second provisional directorship of IPI in 1937, he was in charge of the Public Health Department in Tonekabon. Later, during 1938 till 1940, aside from his IPI duties, Dr. Bahrami also served as the Head of Public Health Administration^[^^15^^]^. 

Since its inauguration, IPI has been the main referral agency by the government in times of major outbreaks caused by infectious diseases. During the provisional directorship of Dr. Bahrami, a couple of such outbreaks occurred and are recorded in the literature. For instance, in 1930s, when a severe epidemic of rinderpest (bovine plague) hit the country and caused extensive deaths in cows, the control of the disease was assigned to IPI, until the arrival of French veterinary microbiologist, Dr. Louis Pierre Joseph Delpy (1899**–**1974), in Iran, who took over the task at the Razi Institute of Iran^[^^16^^]^. According to an article by Dr. Ghodssi^[^^17^^]^, severe cases of dysentery were reported in the military barracks in Tehran in 1937. Under the directorship of Dr. Bahrami, IPI investigated the source of infection, as requested by military officials. Based on the investigations and prepared cultures, the source of the infection was determined as *Shiga* bacillus. Another of such major health threats occurred during the mass exodus of Polish nationals from the Soviet Union and their subsequent arrival in Iran in extremely poor conditions. The first waves of these refugees who were suffering from a multitude of infectious diseases crossed the Iranian borders in 1942. As documented by the capital’s newspaper, *Etelaat*, on September 6^th^, 1942, under the supervision of Dr. Bahrami, IPI was asked to set up medical laboratories for the Polish refugees in order to care for their infections during that summer.

**The final years**

In 1946, Dr. Bahrami became in charge of teaching physics at the Higher College of Hygiene in Tehran, established in order to compensate for the shortage of physicians in Iran. As indicated in his book, published by Tehran University^[^^12^^]^, he was also promoted to Associate Professor at Tehran University where he taught courses in the faculty of Veterinary Medicine. In his final years, he practiced medicine in Reza-Noor Hospital, located at Ghavam-al-Saltaneh Street in central Tehran. Dr. Bahrami died in 1950 due to complications caused by a liver disease at the age 56 and was buried in Zahir al-Dowleh Cemetery in the north of Tehran^[^^6^^]^. Dr. Bahrami left behind four daughters, Gloria, Parvin, Pouran, and Victoria, and two sons, Houshang and Manouchehr^[^^1^^]^.
